# Identifying important breast cancer control strategies in Asia, Latin America and the Middle East/North Africa

**DOI:** 10.1186/1472-6963-11-227

**Published:** 2011-09-20

**Authors:** John FP Bridges, Benjamin O Anderson, Antonio C Buzaid, Abdul R Jazieh, Louis W Niessen, Barri M Blauvelt, David R Buchanan

**Affiliations:** 1 Department of Health Policy and Management, Johns Hopkins Bloomberg School of Public Health, 624 N. Broadway, Baltimore, MD 21205, USA; 2 Department of Surgery, University of Washington, 1959 NE Pacific St, Seattle, WA 98195, USA; 3 Hospital São Jose, Centro Avançado de Oncologia, Rua Martiniano de Carvalho, 951 - Bela Vista, 01321-001, Sao Paulo, Brazil; 4 Department of Oncology, King Abdulaziz Medical City Riyadh, P.O. Box 14691, Riyadh, 11426, Saudi Arabia; 5 Department of International Health, Johns Hopkins Bloomberg School of Public Health, 615 N. Wolfe Street, Baltimore, MD 21205, USA; 6 Health Policy and Practice, Faculty of Medicine and Health Sciences, University of East Anglia, University Drive, Norwich NR4 7TJ, UK; 7 Institute for Global Health, University of Massachusetts, 715 North Pleasant Street, Amherst, MA 01003, USA

## Abstract

**Background:**

Breast cancer is the most frequent cause of cancer death in women worldwide, but global disparities in breast cancer control persist, due to a lack of a comprehensive breast cancer control strategy in many countries.

**Objectives:**

To identify and compare the need for breast cancer control strategies in Asia, Latin America and the Middle East/North Africa and to develop a common framework to guide the development of national breast cancer control strategies.

**Methods:**

Data were derived from open-ended, semi-structured interviews conducted in 2007 with 221 clinicians, policy makers, and patient advocates; stratified across Asia (n = 97), Latin America (n = 46), the Middle East/North Africa (ME/NA) (n = 39) and Australia and Canada (n = 39). Respondents were identified using purposive and snowballing sampling. Interpretation of the data utilized interpretive phenomenological analysis where transcripts and field notes were coded and analyzed and common themes were identified. Analysis of regional variation was conducted based on the frequency of discussion and the writing of the manuscript followed the RATS guidelines.

**Results:**

Analysis revealed four major themes that form the foundation for developing national breast cancer control strategies: 1) building capacity; 2) developing evidence; 3) removing barriers; and 4) promoting advocacy - each specified across five sub-ordinate dimensions. The propensity to discuss most dimensions was similar across regions, but managing advocacy was discussed more frequently (p = 0.004) and organized advocacy was discussed less frequently (p < 0.001) in Australia and Canada.

**Conclusions:**

This unique research identified common themes for the development of breast cancer control strategies, grounded in the experience of local practitioners, policy makers and advocacy leaders across diverse regions. Future research should be aimed at gathering a wider array of experiences, including those of patients.

## Background

Breast cancer ranks as the fifth cause of death from cancer overall, but it is still the most frequent cause of cancer death in women in both developing and developed regions [1]. As such, breast cancer control has become a global imperative, yet global inequities persist [2,3]. Many lesser developed countries in Asia, Latin America, the Middle East and North Africa lack adequate breast cancer services for screening and treatment and experience higher mortality rates compared to more favorable survival from breast cancer in (high-incidence) developed regions [4-6]. This gap will widen further as many recent advances in early detection and treatment remain largely confined to industrialized nations [7] and significant disparities in breast cancer research exist [8]. To address disparities in breast cancer control, a concerted international effort is necessary [9]. Such an effort must be supported and informed by evidence on national priorities for breast cancer control.

The purpose of this study was to identify and compare important breast cancer control strategies in Asia, Latin America and the Middle East/North Africa to develop a common framework as a guide to the development of national breast cancer control strategies. This is an important research question, as little can be found in the literature on the perceived public policy needs of smaller or lower-resourced countries. While several international organizations have documented cancer control strategies or therapeutic guidelines [10,11], this study identified elements of breast cancer control strategy relevant to public policy and clinical practice in the study countries. This was achieved through the use of qualitative research methods to identify strategies relevant to a variety of stakeholders (clinicians, policy makers and patient advocates). The regions covered in this study (Asia, Latin America, and the Middle East/North Africa) account for approximately 60% of the world's population of women and a growing percentage of the incident cases of breast cancer [12]. Australia and Canada were included in the study as controls. Data from these countries helped identify some factors that may have been somewhat neglected in the other countries. Departing from traditional qualitative methods, we interviewed a large number of respondents to facilitate a comparison of the similarities and differences between regions and our controls.

This unique study is the first to highlight the special challenges smaller, lower-resourced countries face to achieve breast cancer control. Our research can be used to inform such countries on the development of breast cancer control strategies that are broader than the creation of clinical guidelines. We also demonstrate the value of qualitative methods in informing public policy.

## Methods

We utilized qualitative research to identify breast cancer needs from the perspective of various stakeholders [13-15], a method well suited to exploratory studies aimed at understanding the needs and experiences of the respondents [16,17]. We utilized a very large number of interviews to gather data on a diverse range of experiences and to facilitate a simple statistical comparison of the propensity to discuss themes by region.

Countries were selected by a group of advisors representing breast cancer experts from across each region with a goal of at least 200 qualified respondents representing medicine, as well as policy and patient advocacy where they existed. Respondents were identified through a combination of purposive and snowball sampling methods [16,18] to ensure that we identified a breadth and depth of experience and knowledge in breast cancer clinical practice, policy making, and patient advocacy from across these diverse regions, based on explicit inclusion criteria (Table 1).

**Table 1:** Inclusion criteria

Respondents	Definition
Medical thought leader	Initial list identification:
	• Individuals involved in breast cancer medicine with a history of significant publications and presentations at major medical and scientific symposia
	• Heads of leading local medical schools and/or research-based teaching hospitals or cancer centers
	• Leaders of local societies relevant to breast cancer medicine or members of national cancer study groups, breast cancer research councils, or tumor boards
	• Individuals with an active and wide publication history or those who had made presentations within the past few years at the American Society of Clinical Oncology (ASCO), St. Gallen, and the San Antonio Breast Cancer Symposium (SABCS)
	The list was then cross-checked to assess if these clinicians were:
	• Heads of local leading medical schools and/or research-based teaching hospitals or cancer centers; and/or
	• Leaders of breast cancer medical or other relevant medical societies responsible for breast cancer medicine; and/or
	• Members of national cancer study groups, breast cancer research councils, and/or national tumor boards.

Policy maker	The selection of policy thought leaders was based on:
	• Information obtained from the Ministries of Health which recommended the appropriate personnel in charge of breast cancer policy, funding, screening, and public education
	• In most cases, nomination and/or validation by the medical thought leaders and/or advocacy leaders.

Patient advocacy leader	Advocacy leader selection came from:
	• Referrals by medical or public policy thought leaders; and/or
	• Independent internet searches and media citations; and/or
	• Other advocacy leaders both domestically and internationally.

Target respondents were initially identified from a literature review, engagement with content experts and via other respondents. All were sent an invitation letter outlining the purpose of the study, the funding source and the potential benefits and risks (which were minimal) of the study. As seen in Table 2 of 375 identified target respondents, 75 did not respond, often due to invalid contact details.

**Table 2:** Respondents and non-respondents by region

	Asia	Latin America	Middle East/North Africa	Australia and Canada	Total
Targets (total)	127	83	67	90	375

**Excluded from analysis**					

No response	14	21	12	28	75

Refused	4	4	3	7	18

Not eligible	15	12	10	16	53

**Included in analysis**					

Physician	27	26	19	10	82

Surgeon	16	3	0	0	19

Hospital manager	22	12	10	6	50

Academic	11	4	6	5	26

Researcher	11	0	3	5	19

Nurse	0	0	0	3	3

Policy maker	4	1	1	4	10

Patient advocate	3	0	3	6	12

All	94	46	42	39	221

All potential respondents were contacted by telephone, and questions were asked to confirm eligibility and to schedule a subsequent complete interview in person or by telephone. A follow-up letter or email was sent within two weeks if no reply was received, with a translation for those target respondents for whom English was not a primary language. If no reply was received within two weeks, or if the email address or letter was not deliverable, the study team attempted calling them by telephone to verify their interest in participation. Upon verification of interest and eligibility, the interview was scheduled and conducted. From initial contact, 53 were not eligible because they did not meet inclusion criteria. A further 18 refused to participate, most often citing a lack of time, although one respondent refused to participate without financial compensation, and four due to industry funding of the study.

The final 221 interviews were conducted and coded by a team of 10 experienced and culturally competent field workers led by a study co-author (BB) who conducted over a quarter of the interviews. No field worker was directly involved in breast cancer nor previously advocated for breast cancer control. Hence, the research took little in the way of bias into the interviews, allowing for a very neutral documentation of the study findings. This said, we acknowledge several possible sources of bias related to the study team. First, countries engaged in internal or external conflict were excluded to ensure the safety of the research team. Second, central and eastern Europe were excluded as another organization was actively recruiting similar study subjects for a study on clinical breast cancer guidelines [9] and the study team did not want the two studies to be confused. Finally, given that English was the common language of the interviewers, they may have inadvertently biased the interpretation and analysis of responses towards Western concepts. The focus on English may also have led to a biased sample as verification of eligibility often relied upon publication in peer reviewed journals and review of conference proceedings, both of which are biased towards English-language publications. The interpretation of the study results, and the subsequent identification of the taxonomy of key themes, was developed by the lead author (JB) in conjunction with other investigators on the study. This coding was done with all identifiers removed, with the exception of region, which facilitated an equal weighting of the data from all respondents. In addition, an external panel of non-compensated experts in breast cancer reviewed the analysis, results and conclusions.

Prior to the start of each interview, respondents were again informed about the purpose of the study, the source of the study funding, the risks and benefits of participating and that, while the results of the study may be shared in the public domain and respondents may be quoted verbatim, the content of the interview would be kept anonymous and confidential. Given the nature of the study, the minimal risks involved, and the anonymous nature of the study data, the study was exempt from any formal ethics review.

Interviews were conducted using a standardized interview protocol that had been extensively piloted. Interviews began with a 'grand tour' question: *"What are the most significant medical or other challenges that you see yourself facing in the next few years with regards to breast cancer?" *Specific probes were used to gain more detailed facets of best practices, support, collaborative opportunities and involvement of other stakeholders. Interviews lasting 1 to 1.5 hours were conducted in person or by telephone in English or in the respondent's native language by trained fieldworkers with extensive experience conducting medical/scientific interviews in the relevant countries. When conducted in the interviewee's native language, the field notes were immediately translated by the interviewer fluent in both languages.

Data were analyzed both qualitatively and semi-quantitatively to aid both identification and comparison of themes respectively. The identification of themes utilized Interpretive Phenomenological Analysis in order to capture respondents' direct experience with breast cancer control in their own countries [19]. Analysis was facilitated through the construction of a standardized coding protocol that was iteratively developed by the research team.

Coded transcripts and field notes were then compared, and a taxonomy consisting of themes and dimensions was developed without reference to any pre-existing conceptualization of breast cancer control strategies. Consistent with the method of analysis [19], representative quotes were extracted to illustrate the key themes. We did not attribute the quotes to any region or respondent for two important reasons. First, as part of the consent process, respondents were informed that they would not be linked to their responses. Second, quotes were identified for their generality, and hence if we attributed the quotes even to regions readers would be compelled to find qualitative differences, rather the commonalities in the factors identified. This said, we subsequently used a more robust statistical approach to explore variations in the propensity to discuss the themes that we identified.

We also used semi-quantitative analysis to compare responses across regions. Here we estimated the propensity, calculated as a percentage of respondents, for discussion of each of the final coded themes by region [20]. We did not account for multiple references to a theme within an interview with a single respondent (i.e. we map the propensity of referring to each item at least once within an interview). To test for differences in these propensities of discussion of the 20 dimensions (measured as a percentage of respondents in each region who discussed the dimension) ANOVA was used, with the null hypotheses that the percentage of respondents discussing any particular dimension was identical. In cases where the ANOVA identified a significant difference across the regions (p < 0.05) for a particular dimension, we subsequently conducted a multiple pair-wise comparisons test using the Mariscuillo procedure [21,22] to further compare differences across the four regions for that dimension. Based on this test, we identified which of the four regions were statistically different from each other region in terms of the percentage of respondents referring to the dimension.

Several techniques were used to ensure that data analysis was systematic and verifiable, as recommended for qualitative research [18,19]. Triangulation methods were used in the early development of the study protocol, during the pilot phase, during the development of the coding system and for interpretation of the data. During the field work, data were prospectively coded using an evolving coding manual. The taxonomy was developed by consensus, informed by qualitative and quantitative analysis of the data. Early results were presented at several workshops at a number of international breast cancer meetings, with many of the study respondents and other world leaders in breast cancer participating. During these meetings, early stage results were commented upon, with many of the participants contributing to the interpretation of the data. Finally, we utilized the RATS guidelines to ensure all relevant information was included in the manuscript [23,24].

## Results

**Figure 1:**
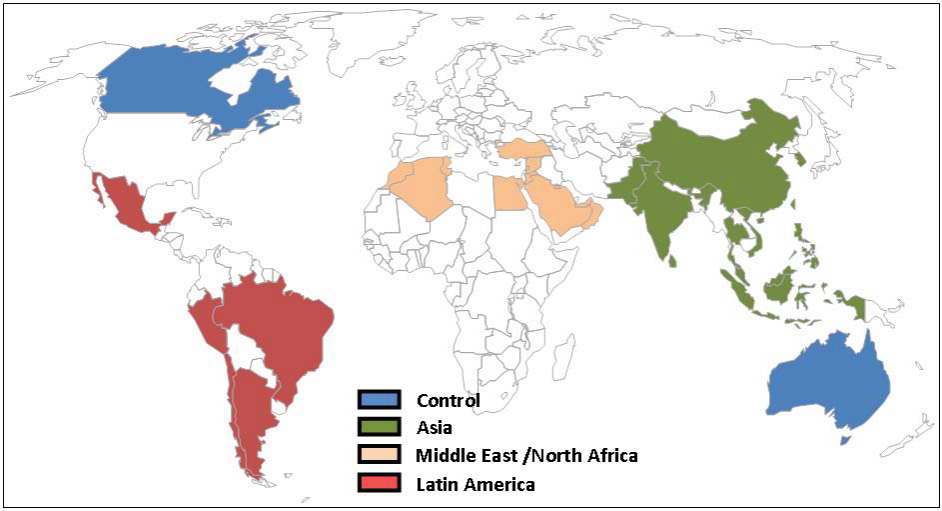
**Countries included in the analysis**. Figure 1 identifies the countries included in the study. Countries included in the study were (with number of respondents per country presented in parentheses): Asia: South Korea (20); China (19); Taiwan (16); India (13); Thailand (6); Malaysia (5); Philippines (5); Indonesia (5); Singapore (4); Vietnam (1). Latin America: Mexico (19); Brazil (19); Argentina (6); Peru (1); Chile (1). Middle East/North Africa: Turkey (20); Egypt (5); UAE (3); Lebanon (3); Pakistan (3); Saudi Arabia (2); Jordan (1); Syria (1); Oman (1); Morocco (1); Tunisia (1); Algeria (1). Control group: Australia (17); Canada (22).

As previously seen in Table 2 the final sample of respondents included a broad range of stakeholders with relevant experiences and understanding of breast cancer control, including physicians, surgeons, hospital managers, academics, researchers, nurses, policy makers, and patient advocates. The total number of respondents was 221 spanning 29 countries (see Figure 1).

### Taxonomy of results

Based on the analysis of the interview data, national breast cancer control strategies can be described across four broad themes: *building capacity*, *developing evidence*, *removing barriers*, and *promoting advocacy*. Table 3 summarizes these themes and the five dimensions contained in each theme.

**Table 3:** Themes and dimensions

Theme	Dimension	Concepts
Building capacity	Science and research	Capacity for basic and clinical science; funding for clinical research; trained nurses/staff for clinical research.
	
	Skilled nurses	Capacity to train nurses for patient care and patient education and to provide qualified nursing support.
	
	Research infrastructure	Capacity for clinical research; need for research laboratories; need for research equipment.
	
	National statistics	Capacity to investigate local incidence and characteristics of breast cancer; IT capacity to support national registries and research.
	
	Public education	Capacity for national public education and awareness campaigns on breast cancer and screening.

Developing evidence	Study of local etiology	Evidence identifying differences between local patients and those in the UK/USA; younger women; more aggressive tumor morphology.
	
	Personalized therapy	Evidence to promote personalized therapy; includes using genetic targets to tailor treatment.
	
	Developing guidelines	Research in local populations and other evidence to inform local guidelines and policies; national treatment guidelines and coordination.
	
	International networks	Connecting to evidence internationally; keeping up to date; global research programs, networking and education opportunities.
	
	Local communication	Communicating evidence across stakeholders; improved communications between/within institutions and across institutions within areas or the country.

Removing barriers	Out-of-pocket costs	Barriers due to out-of-pocket expenses paid for by the patient.
	
	Disparities in access	Barriers for underserved and rural populations.
	
	High cost to payers	Barriers to reimbursing high-cost treatments.
	
	Early detection	Barriers to accessing earlier detection.
	
	Reimbursement	Barriers to accessing therapies that are not yet proven to be cost effective.

Promoting advocacy	Patient empowerment	Strategies to empower patients/patient groups and inform consumers.
	
	Managing survivorship	Support increased focus on survivors; long-term side-effects and quality of life.
	
	Quality of life	Support increased focus in research and practice on quality of life.
	
	Metastatic disease	Support for the management of metastatic disease.
	
	Organized advocacy	Support for leadership and staffing of advocacy groups; communication between advocacy groups.

### Theme 1: Building Capacity

The theme of *building capacity *represents dimensions such as developing the capacity for *science and research*, the need for *skilled nurses*, funding and development of *research infrastructure*, collecting and disseminating *national statistics*, and *public education *of the issues associated with breast cancer. Representative quotes for the theme of *building capacity *are presented in Table 4.

**Table 4:** Dimensions of building capacity

Dimension	Representative quote
Science and research	"*Research is our major issue. We are getting farther and farther behind the rest of the world*." [Physician from Middle East/North Africa]

Skilled nurses	*"We depend upon employing nurses from other countries to fill our needs. This hampers our ability to advance breast cancer care of our own people, by our own people*." [Surgeon from Middle East/North Africa]

Research infrastructure	*"How can we do basic research when there will be no jobs for these PhDs upon completing years of study, or when for the same investment of their time and talents, they will find more lucrative jobs as physicians." *[Researcher from Australia]

National statistics	*"Without a long term commitment to developing data at a national level, we cannot develop the appropriate guidelines and policies appropriate to our own population."* [Physician from Latin American]

Public education	*"Public education, especially on the importance of early detection, is key."* [Policy maker from Asia]

Capacity for *science and research *was commonly addressed in terms of insufficient funding of clinical science and a lack of trained human resources to support basic science and clinical research. As one respondent stated, the responsibility may fall on the individual investigator to "...*educate nurses and other staff to do research, because the government will not do this or fund this*". Respondents discussed the need to build a critical mass of local basic and clinical research and identified roles for both national governments and the international community to support research activity.

*Skilled nurses *were cited in each region in terms of the need for more trained nurses to support breast cancer patient care, education, and research, as well as the need to be able to retain such staff. As one respondent put it, currently skilled nurses are "...outnumbered in terms of patients. In oncology, the workforce, particularly in nursing, is declining".

*Research infrastructure *emerged as another dimension, in terms of fostering a supportive environment for clinical and bench-top research. To support well-functioning and productive research enterprises, more funding for equipment and staff is also needed. In some countries, universities do not offer the training required for breast cancer research. Many countries cited acute shortage of PhD-qualified researchers as hampering capacity for breast cancer research.

Respondents identified a broad need for *national statistics*, a dimension covering a range of parallel issues, including the need for benchmarking, national registries, epidemiological statistics, and capacity for information technology and data management. National statistics require national investments in robust data collection and management capacity to identify and explain potential differences in the occurrence of breast cancer locally, in particular, whether there are disparities in the incidence and etiology of breast cancer in different populations.

Building capacity for *public education *to promote widespread awareness of breast cancer was noted as an important priority. Countries need to establish consistent messaging that goes beyond at-risk populations to generate widespread knowledge of breast cancer in the general population. For example, one country had already incorporated breast cancer awareness into public media campaigns and school programs to demonstrate "...*how to do breast self-examination and to make this at least a monthly routine*".

The propensity to discuss issues of *building capacity *was relatively consistent across the regions (Table 5), with the exception of the propensity to discuss research infrastructure (p = 0.01) and national statistics (p = 0.003). With regards to discussing research infrastructure, the propensity was highest in Latin America (59%) and lowest in Asia (30%), with Middle East/North Africa (31%) and Canada/Australia (41%) falling in between. The need for national statistics was discussed most prominently in Middle East/North Africa (51%) and Canada/Australia (56%), at a rate higher than in Latin America (37%) and much higher than in Asia (27%).

**Table 5:** Propensity to discuss each dimension by region

	Asia N = 97	Latin Am N = 46	ME/NA N = 39	Aus/Can N = 39	p value
**Building capacity (%)**					

Science and research	51.5	50.0	56.4	46.2	0.84

Skilled nurses	50.5	41.3	51.3	38.5	0.48

Research infrastructure	29.9^a^	58.7^b^	30.8^a, b^	41.0^a, b^	0.01

National statistics	26.8^a^	37.0^a, b^	51.3^a, b^	56.4^b^	0.003

Public education	42.3	47.8	46.2	33.3	0.56

**Developing evidence (%)**					

Study of local etiology	23.7	37.0	28.2	33.3	0.38

Personalized therapy	36.1^a^	82.6^b^	20.5^a^	48.7^a^	< 0.001

Developing guidelines	23.7^a^	47.8^b^	12.8^a^	51.3^b^	< 0.001

International networks	52.6^a, b^	63.0^b^	30.8^a^	66.7^b^	0.01

Local communication	36.1^a^	34.8^a^	10.3^b^	56.4^a^	< 0.001

**Removing barriers (%)**					

Out-of-pocket costs	38.1^a, b^	47.8^b^	10.3^c^	20.5^a, c^	< 0.001

Disparities in access	37.1^a, c^	69.6^b^	23.1^a^	53.8^b, c^	< 0.001

High cost to payers	42.3	56.5	38.5	53.8	0.22

Early detection	45.4	50.0	46.2	41.0	0.88

Reimbursement	43.3^a, b^	45.7^a, b^	28.2^b^	61.5^a^	0.03

**Promoting advocacy (%)**					

Patient empowerment	26.8^a^	39.1^a, b^	23.1^a, b^	53.8^b^	0.008

Managing survivorship	12.4^a^	17.4^a^	0.0^b^	33.3^a^	0.004

Quality of life	19.6	28.3	20.5	33.3	0.31

Metastatic disease	36.1	26.1	33.3	28.2	0.63

Organized advocacy	13.4^a, c^	43.5^b^	28.2^c^	5.1^a^	< 0.001

Based on the Mariscuillo multiple comparisons procedure [21,22], common superscripts ^a, b, c ^reflect paired comparisons that are not statistically different, while pairs that are statistically different do not share the same superscript.

### Theme 2: Developing evidence

The second theme that emerged from our discussions with the key informants was centered on *developing evidence*, including dimensions such as the *study of local etiology*, developing suitable approaches to *personalized therapy*, *developing guidelines*, fostering *international networks *and promoting *local communication *to enable sharing of and the ability to build upon collective experience. Representative quotes relating to the dimensions of developing evidence, are presented in Table 6 and are discussed in full below.

**Table 6:** Dimensions of developing evidence

Dimension	Representative quote
Study of local etiology	"*Asian women get breast cancer earlier than US women. We need to know why*." [Hospital manager from Asia] "*We are seeing breast cancer in women 10 years earlier than Caucasians*." [Surgeon from Asia]

Personalized therapy	"*We need to individualize therapy; however, we have to follow a cook book approach or funds are cut*." [Physician from Canada]

Developing guidelines	*"Breast cancer in developing countries is not the same as in the West. We need non-Western clinical protocols to address the need for screening among younger cohorts*." [Hospital manager from Middle East/North Africa]

International networks	"*Currently, we have to wait every six months to meet in international conferences to exchange information with colleagues. I would like to find the opportunity where I can discuss clinical issues more often with colleagues."* [Academic from Asia]

Local communication	*"We should be continuously discussing and comparing notes within our own group as well as with other cancer centers, we should be pooling and building on our collective local knowledge"* [Hospital Manager from Latin American]

*Study of local etiology *focused on the need to collect and analyze data in a way that identifies variations in disease etiology, such as higher proportions of breast cancer diagnosis among younger women, more aggressive disease, and differences in tumor morphology of breast cancer in the respondents' home country.

*Personalized therapy *was generally expressed as a need to focus on individual patient treatment, support and other care, including utilization of targeted therapies. Interestingly, there was significant regional variation in terms of the frequency with which personalized therapy was discussed. In Latin America, for instance, there was a great deal of general interest in personalized therapy. In the Middle East/North Africa, interest in personalized approaches was motivated by the relatively high number of women who present with late stage breast cancer.

*Developing guidelines *at the national level was a major dimension of the *developing evidence theme *and is related to both the need for national statistics and the coordination of practitioners, researchers, advocates and policymakers. Some respondents stressed that guidelines were nonexistent, unclear or incomplete, or lacked appropriate stakeholder representation during development. Often guidelines were merely copied from international standards, without reference to local needs, circumstances, or resources.

The *international networks *dimension of the developing evidence theme reflected the need for dynamic, continuous communication between local researchers/clinicians and the international community. Such two-way communications can be facilitated by global research programs and international training programs. As one respondent put it, there is a *"...need for more research with international exposure"*. While countries in South America had benefited from such networks, respondents from the Middle East/North Africa were significantly less likely to be engaged in collaborative trials from outside of the region, and to send research fellows to USA and EU.

*Local communication *related to the need for better communication about research and practice within multi-disciplinary teams, both within single organizations and across distinct organizations within the same area or nation. Improved local communication, especially on research activities, can stimulate and reinforce a culture of clinical and basic research. This need for improved communication was mentioned even by those countries with the highest level of commitment to breast cancer research.

The propensity to discuss the *study of local etiology *was relatively common across the regions (see Table 5), but significant variations existed in the propensity to discuss the other dimensions: *personalized therapy *(p < 0.001), *developing guidelines *(p < 0.001), and *communication *(p < 0.001). *Personalized therapy *was discussed among most respondents in Latin America (83%), and much less in Asia (36%), Middle East/North Africa (21%), and even Canada/Australia (49%). The propensity to discuss *developing guidelines *was similar between Latin America (48%) and Canada/Australia (51%), but was discussed less often in Asia (24%) and far less often in the Middle East/North Africa (13%). Those respondents in the Middle East/North Africa (31%) discussed *international networks *significantly less than those in Latin America (63%) and Australia (67%), with respondents in Asia (53%) falling in between. Finally, the focus on local *communication *in Asia and Latin America was almost identical (36% and 35%, respectively), and were not statistically different than those of Australia/Canada (56%). The results for Middle East/North Africa were significantly different from all the other groups, with *communication *discussed by only 10% of respondents.

### Theme 3: Removing barriers

The third theme was *removing barriers *to cancer control, including dimensions related to the high *out-of-pocket costs *faced by many women with breast cancer, addressing *disparities in access*, the *high cost to payers *of breast cancer, the need for *early detection *of breast cancer, and issues of cost-effectiveness that can act as a barrier to the *reimbursement *of breast cancer treatments. Selected quotes from respondents relating to the dimension of *removing barriers *are presented in Table 7 and are discussed as follows.

**Table 7:** Dimensions of removing barriers

Dimension	Representative quote
Out-of-pocket costs	"*The government hospital will pay for a large portion of treatment, but women have to pay for the initial mammography and biopsy. They have to have a positive diagnosis before the government will start paying. That is a huge deterrent."* [Policy maker from Middle East/North Africa]

Disparities in access	*"Managing breast cancer among underserved populations is poor across the board. These populations are undereducated and the government is not focusing on them."* [Patient advocate from Australia]

High cost to payers	*"Our government will not pay for breast cancer for the majority of the population." *[Surgeon from Latin America]

Early detection	*"We need better strategies to encourage women of all ages to regularly practice self-detection. We estimate fewer than 5% of women do this." *[Surgeon from Asia]

Reimbursement	*"The most significant challenge is being able to demonstrate the cost-benefit of breast cancer therapy in an objective way. There is a particular issue around the affordability of drugs and associated pathology testing--particularly new drugs."* [Hospital manager from Canada]

The dimension of *out-of-pocket costs *mainly reflects barriers to accessing care by patients who bear high costs of care, not only directly with payments and co-payments but also indirectly through travel costs or lost earnings, for example. For the majority of the countries examined, there is a lack of or insufficient insurance from either private or public sectors. Where it is available, many procedures and tests are not covered, or not covered adequately. The inability of the average woman with breast cancer in these countries to pay for diagnosis, prevention, or treatment results in women presenting to hospitals with very late stages of breast cancer, when prognosis is poorest.

The dimension of *disparities in access *highlights the plight of poor, rural, and other underserved populations in many countries who often have limited or no access to screening, outpatient services, and breast cancer management. A lack of resources was often cited as the main reason for these disparities, but issues such as distance, language, culture, and stigma also play a role. As one respondent put it, *"many women in rural areas have to travel and be away from home in order to access care." *Many populations lack awareness of even basic breast cancer services such as screening, highlighting a need for *"... underserved population[s] to be educated regarding breast cancer"*.

The *high cost to payers *dimension reflected difficulties in reimbursing new high-cost treatments as well as covering basic care (e.g. screening and prevention) to a growing population of patients. It highlighted insufficient reimbursement and funding by governments, private insurance and other health plans. As both technical capacity and patient needs grow, the *"...biggest challenge in the next few years is funding the high cost of breast cancer drugs*".

*Early detection *was highlighted as a need for eliminating barriers to clinical and self-examination, including screening among younger women, and the need for more aggressive national policies and programs for early detection and screening. Even though many of the countries that participated in our study have national awareness programs, these do not reach a wide audience and lead to disparities in early detection.

Barriers to access were further discussed in the context of inability to demonstrate cost-effectiveness of new technologies and therapies, which limits reimbursement of costs therein. Respondents indicated that this is an emerging problem, particularly for high-cost, targeted therapies. As one clinician remarked, *"The most significant challenge is being able to demonstrate the cost-benefit of breast cancer therapy in an objective way"*.

Regions were relatively similar in the propensity to discuss high costs to payers and early detection (see Table 5). Significant variation was identified on the dimensions of *out-of-pocket costs *(p < 0.001) and *disparities in access *(p < 0.001), and to a lesser extent for the *reimbursement *dimension (p = 0.03). Patient *out-of-pocket costs *were discussed most in Asia (38%) and Latin America (48%), significantly more than in Middle East/North Africa (10%). The propensity to discuss the disparities* in access *dimension was greatest in Latin America (70%), a figure that was similar to that in Australia/Canada (54%) but significantly greater than that in Asia (37%) and Middle East/North Africa (23%). The dimension of *reimbursement*, covering issues of cost-effectiveness, was most often discussed in Australia/Canada (62%) where formal technology assessment procedures require cost-effectiveness before reimbursement, but the rate of discussion was statistically similar for Asia (43%) and Latin America (46%), although it was significantly lower in Middle East/North Africa (28%).

### Theme 4: Promoting advocacy

The fourth theme of our comprehensive framework for national breast cancer control was *promoting advocacy*, which covers issues such as promoting *patient empowerment*, *managing survivorship*, the *quality of life *of women with breast cancer, the need to focus on *metastatic disease*, and the need for *organized advocacy *efforts in breast cancer. Selected quotes for the dimensions of promoting advocacy are presented in Table 8.

**Table 8:** Dimensions of promoting advocacy

Dimension	Representative quote
Patient empowerment	*"We are challenged by women who are younger and better educated. They want to know everything and be involved with all decisions [about their breast cancer]." *[Policy maker from Australia]

Managing survivorship	"*We need more nurses and trained staff to take on the challenges related to the growing number of breast cancer survivors and the increase of side effects including the long-term effects of worrying and on the family*" [Hospital manager from Latin America]

Quality of life	"*Quality of life is important because breast cancer patients can live for a long time and their emotional well being is important"*. [Patient advocate from Canada] *"We use quality-of-life protocols derived from western countries, however there are cultural differences that need to be incorporated*." [Physician from Asia]

Metastatic disease	"*Women present for the first time at a late stage of disease - often metastatic*" [Physician from Middle East/North Africa]

Organized advocacy	*"Our government is very concerned about national breast cancer advocacy - if it gains power, then the government not only will have to support it, but it will have to also pay attention to other cancer groups." *[Patient advocate from Asia]

A prominent dimension of promoting advocacy was *patient empowerment*. Respondents highlighted the need for two-way communication between the healthcare system and patients and patient groups, and for patients to become increasingly involved in all aspects of decision-making. Respondents stressed that such communication needs to be tailored to reach groups with diverse backgrounds. Respondents also highlighted a role for improved patient involvement in underserved populations as a means by which to precipitate change.

The need for advocacy to support the emerging issue of *managing survivorship *was limited to certain regions and respondents, due to the small number of advocates and to the fact that many countries are still struggling with providing primary care or basic screening. Yet this minority of respondents strongly emphasized that more focus is needed on survivors in both research and practice. Specifically, more emphasis needs to be placed on long-term side-effects, the ability of primary care providers to manage these survivors, and the quality of life that survivors experience.

The *quality of life *of those with breast cancer also emerged as an important dimension of the breast cancer advocacy theme, in terms of both research and practice. Notably, physician respondents in most countries admitted to not having the time nor the training and resources to counsel patients on what to expect during or after surgery and/or chemotherapy. As one respondent put it, *"We don't have time to discuss issues such as quality of life during and after breast cancer treatment."*

Improved patient advocacy to highlight the plight of patients with *metastatic disease *was also highlighted by many respondents in each region, mainly in the context of necessity of resources to provide palliative care. Respondents indicated that this was of greater concern when there were an increasing number of survivors or when women delayed seeking medical care and presented for the first time at a later stage of disease. Access to narcotics for pain control is a particularly common issue in countries with limited resources. Another concern expressed was the often prohibitive cost versus benefits of treatment options in metastatic disease.

Given the general lack of *organized advocacy *in many of the regions studied, respondents indicated the need for national strategies to improve organized advocacy efforts. While advocacy normally starts at a 'grass-roots' level, respondents stressed the importance of advocacy at the national level, where they could achieve significant impact. More specifically, respondents recognized that advocacy is needed to represent consumers and patients in ways that medical thought leaders and policy makers cannot. One respondent lamented the 'Catch-22' situation arising from a lack of advocacy, stating that the *"...government doesn't want advocacy as breast cancer is not its priority, but without national advocacy, who will represent the women?"*

For the theme of promoting advocacy, the propensity to discuss the *quality-of-life *and *metastatic disease *dimensions were statistically similar across the four regions (see Table 5). Differences were identified for the dimensions of *patient empowerment *(p = 0.008), *managing survivorship *(p = 0.004) and *organized advocacy *(p < 0.001). The propensity to discuss *patient empowerment *was statistically similar across Asia (27%), Latin America (39%) and Middle East/North Africa (23%), while it was most often discussed in Australia/Canada (54%). The dimension of *managing survivorship *was seldom discussed in the emerging regions (0-17%), but was discussed by a third of respondents in Australia/Canada. Finally, the need for *organized advocacy *efforts was discussed most in Latin America (44%), less in Middle East/North Africa (28%) and Asia (13%) and rarely Australia/Canada (5%) where breast cancer advocacy groups and networks already exist at the federal level.

## Discussion

**Figure 2:**
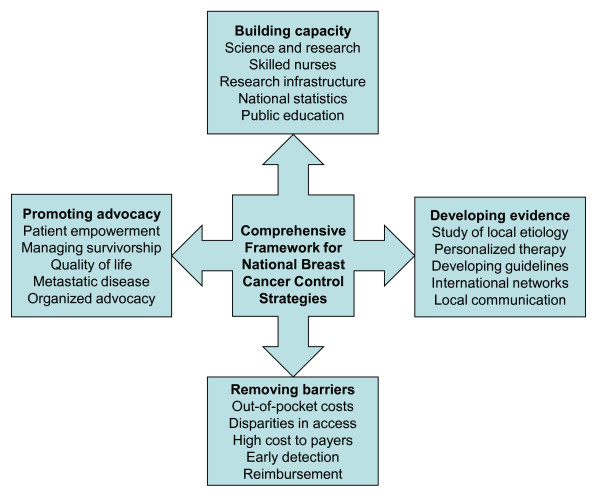
**The comprehensive framework for national breast cancer control strategies**. Figure 2 presents the comprehensive framework for national breast cancer control strategies.

While most countries have developed clinical guidelines for the prevention and treatment of breast cancer, few have developed comprehensive breast cancer control plans. The themes and strategies identified in this study provide countries with a template for developing national breast cancer control plans or, potentially, a mechanism for the assessment of existing control strategies. Figure 2 presents our taxonomy for comprehensive breast cancer control implementation, which can be contrasted to that of the WHO [10]. We find that such a framework must build capacity, take into account developing evidence, remove barriers, and promote patient advocacy. The taxonomy outlining the dimensions of these four themes offers a useful template that can foster both local and global action. As such, this study is an important step towards developing an evidence-based approach to assessing preparedness for implementing comprehensive breast cancer control strategies [7,9].

Our results have several commonalities with the WHO guidelines for national cancer programs that span six dimensions: prevention, early detection, diagnosis/treatment, pain relief/palliative care, cancer control research and surveillance [10]. This said, our conceptualization has several unique areas (for example, personalized therapy, reimbursement, managing survivorship and organized advocacy) not present in the WHO model. Likewise, as a comprehensive approach, our taxonomy does demarcate between stages of disease progression (a key feature of the WHO model), rather than treating breast cancer control as a single entity. Further, we draw no distinctions between the types of strategies that should be used in countries with low, middle, and high levels of resources. Our findings indicate that those developing and implementing a comprehensive breast cancer control strategy should have a single vision in mind, irrespective of country size or level of development. While it is certain that some countries lack the resources to implement such a plan, focus on just a limited number of factors may lead to imbalances (for example, screening is not useful if treatments are not available/affordable and there is not capacity to care for patients). This finding contrasts with existing calls for cancer control strategies that are conditioned on the level of resources available [25].

Our call for a more comprehensive approach to cancer control is not unique. In a recent review on the development of cancer control in developing countries, Hanna and Kangolle [26] call for a model that spans prevention, early detection, diagnosis/treatment, and palliation. Their model focuses around structure, process, and outcomes and has some commonalities with ours, including capacity for research, improved national data, and an improved role of economic evaluation (a key element in addressing the high cost to payers). They too call for more local communication and international collaboration.

A full discussion of the necessary implementation strategies needed for comprehensive breast cancer control is beyond the scope of this paper and detailed strategies are provided elsewhere [10]. Furthermore, the development of a comprehensive approach can draw upon a host of lessons learned from international experience [27].

Just as cancer care is complex and requires coordination [28], so does a national cancer control program. As see in Figure 2, our comprehensive framework for national breast cancer control requires activities in several different areas (building capacity, developing evidence, removing barriers, and promoting advocacy). The simultaneous implementation of such a framework would require dedicated resources for management and stakeholder engagement, and a great deal of political will.

The adoption of a national breast cancer control program in Australia was facilitated by the creation of a National Breast Cancer Center in 1995 that utilized a continuous quality improvement approach to enhancing all aspects of the breast cancer experience [29]. Through the creation/consolidation of data and engagement with stakeholders, the National Breast Cancer Center has identified priority areas of need and developed resources to support interventions. Some of its activities are similar to areas identified in our model, including public awareness, capacity for science and research, developing guidelines and promoting the use of specialty breast cancer nurses [29].

In countries with low levels of resources, national cancer control can be achieved without the creation of new institutions, but through better communication and coordination of existing centers (or what is referred to as local communication in our model). Such a model has been used to coordinate a general cancer control program in Uganda that has attracted international collaborations (also a factor in our model) [30].

It is important to note several limitations in the study. First, the study was conducted among thought leaders from a limited set of countries, and findings may have differed if other countries had been included. Second, our findings may have been influenced by our own frame of reference and preconceptions, although several standard qualitative research techniques were used to mitigate this. Third, clinicians dominated our sample due mainly to predominance of medical influence in breast cancer decision-making, and also due to the significant lack of policy and national advocacy leaders specifically in breast cancer in these lower resourced countries. Finally, we also recognize that the views of our respondents may change over time.

While our framework offers a template for countries around the world to assess their preparedness to address the challenges of breast cancer, additional research is needed to validate our taxonomy. Such validation would require a more robust consideration of the elements of the framework, including an assessment of the feasibility and effectiveness of the individual strategies. Upon validation, we envision that our framework can be used to make detailed comparisons across countries and to develop comprehensive evidence-based policy strategies for breast cancer management. This would require the translation of the elements of our model (Figure 2) into indicators that could be assessed as part of a national breast cancer control checklist.

## Conclusions

Our model may promote more comprehensive and sensitive measurement of the challenges presented, so that the themes and dimensions identified in lessening breast cancer-related morbidity and mortality in these regions may be adequately assessed and developed. Moreover, the identification of needs, challenges and trends may help clinicians, researchers, policy leaders and advocates plan effective interventions that focus on previously overlooked areas specific to breast cancer control. While our framework offers a template to assess preparedness to address the challenges of breast cancer, additional research is needed to validate the taxonomy. Upon validation, we envision that our framework can be used to make detailed cross-country comparisons and to develop evidence-based policy strategies for breast cancer control.

## Competing interests

The authors declare that they have no competing interests.

## Authors' contributions

JB led the analysis and interpretation of the data and drafting of the manuscript. BO, AB, and AJ guided the conceptualization of this study and preparation of the manuscript. LN assisted in analysis and preparation of the manuscript. BB and BD were involved in the conception and design and acquisition of data, interpretation of the data and reviewing the manuscript. All authors read and approved the final manuscript.

## Pre-publication history

The pre-publication history for this paper can be accessed here:

http://www.biomedcentral.com/1472-6963/11/227/prepub
